# Direct observation and catalytic role of mediator atom in 2D materials

**DOI:** 10.1126/sciadv.aba4942

**Published:** 2020-06-10

**Authors:** Gun-Do Lee, Alex W. Robertson, Sungwoo Lee, Yung-Chang Lin, Jeong-Wook Oh, Hwanyeol Park, Young-Chang Joo, Euijoon Yoon, Kazu Suenaga, Jamie H. Warner, Christopher P. Ewels

**Affiliations:** 1 Department of Materials Science and Engineering, Seoul National University, Seoul, Republic of Korea.; 2 Research Institute of Advanced Materials, Seoul National University, Seoul, Republic of Korea.; 3 Department of Materials, University of Oxford, Parks Road, Oxford OX1 3PH, UK.; 4 National Institute of Advanced Industrial Science and Technology (AIST), Tsukuba 305-8565, Japan.; 5 Department of Chemistry, Seoul National University, Seoul, Republic of Korea.; 6 Memory Thin Film Technology Team, Giheung Hwaseong Complex, Samsung Electronics, 445-701, Republic of Korea.; 7 Department of Mechanical Engineering, University of Texas at Austin, 204 Dean Keeton Street, Austin, TX 78712, USA.; 8 Institut des Matériaux Jean Rouxel (IMN), Université de Nantes, CNRS UMR 6502, 2 Rue de la Houssinière, F-44322 Nantes, France.

## Abstract

The structural transformations of graphene defects have been extensively researched through aberration-corrected transmission electron microscopy (AC-TEM) and theoretical calculations. For a long time, a core concept in understanding the structural evolution of graphene defects has been the Stone-Thrower-Wales (STW)–type bond rotation. In this study, we show that undercoordinated atoms induce bond formation and breaking, with much lower energy barriers than the STW-type bond rotation. We refer to them as mediator atoms due to their mediating role in the breaking and forming of bonds. Here, we report the direct observation of mediator atoms in graphene defect structures using AC-TEM and annular dark-field scanning TEM (ADF-STEM) and explain their catalytic role by tight-binding molecular dynamics (TBMD) simulations and image simulations based on density functional theory (DFT) calculations. The study of mediator atoms will pave a new way for understanding not only defect transformation but also the growth mechanisms in two-dimensional materials.

## Teaser

Mediator atoms catalyse bond formation and breaking in graphene and its defects.

## INTRODUCTION

The simplicity of graphene’s two-dimensional (2D), single-element structure makes it the perfect model system for defect study, and as such, the structural transformations of graphene defects have been extensively researched through aberration-corrected transmission electron microscopy (AC-TEM) (*1*–*4*) and theoretical calculations (*5*–*9*). The structural evolution of graphene defects has been explained by the Stone-Thrower-Wales (STW)–type bond rotation for a long time (*3*, *4*, *6*–*8*). While STW rotations have a relatively large activation energy (*10*), it has been reported by simulation studies that surface carbon adatoms may weaken the bond strength of surrounding carbon atoms and thus lower the energy barrier for STW-type bond rotations in fullerenes (*11*–*13*) and carbon nanotubes (*14*). These important early studies focused on single-defect formation and annihilation events and specifically on the role of single isolated adatoms. The concepts have yet to be explored in a broader context to fully assess the potential importance of catalyzed bond rotation processes in general 2D lattice deformation and reconstruction processes. It is also known that adatoms are generated under electron irradiation in AC-TEM of graphene or as contaminants in the growth and transfer of graphene (*2*, *15*, *16*). These adatoms are seen to form a bridge-like structure on the graphene surface under AC-TEM (*15*), and it has also been reported that bridging atoms can be found in reconstructions of vacancies, especially odd-numbered vacancies (*2*). Such atoms have natural catalytic effects due to their undercoordination compared to bulk sp^2^-bonded carbon atoms and can thus catalyze the formation and breaking of bonds in graphene, but their precise role and observation in common compound defects in graphene remain unclear. Here, we call such a catalyzing atom a “mediator atom,” because it mediates the bond-switching mechanism. Defect species in graphene and related 2D materials are complex in reality and can include prismatic dislocation loops, grain boundaries, vacancy aggregates, and other extended lattice damage, and it is important to explore the potential for restructuring and self-healing in these cases through mediator atoms. Such complexes may also serve as sources and drains of mediator atom species. Here, we present a comprehensive study into the role of the mediator atom using experimental TEM images as the foundation for a rigorous modeling framework to describe the mediator atom’s formation, dynamics, and quenching in 2D materials.

## RESULTS

**Fig. 1:**
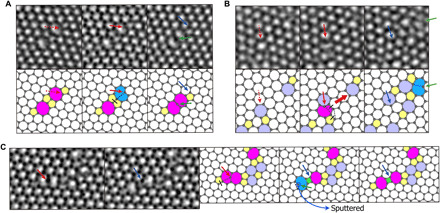
Mediator atom formation from carbon adatom inclusion and subsequent mediator atom–induced structural change. (**A** and **B**) Formation of mediator atom from an incoming adatom and subsequent mediator atom–induced change of the defect structure. (**C**) Mediator atom–induced structural change and its sputtering. Dotted red arrows indicate the position at which the adatom will sit at the following image. Red arrows indicate the position of the adatom. Blue solid arrows indicate atoms forming an sp^2^ bond, which were adatoms before. Green arrows indicate newly formed mediator atoms. Black dotted lines and black solid bars indicate the forming and breaking of bonds, respectively. A red thick arrow in (B) indicates the propagation direction of the mediator atom. The process of structural change by mediator atom in (B) is suggested in detail in fig. S1.

We find that mediator atoms are typically generated from an adatom or vacancy. In the first case, we observe the mediator atom created when an adatom is caught in graphene defects. On pristine graphene, the carbon adatom migrates with a barrier of 0.3 to 0.5 eV (*17*, *18*), which, at room temperature, translates into 10^4^ to 10^8^ hops per second (assuming Arrhenius first-order hopping with a 10^13^-Hz Debye frequency attempt rate). This extremely high mobility means that carbon adatoms are essentially impossible to observe in an electron microscope. However, we have observed adatoms captured in defects; Figure 1 (A and B) shows the inclusion and fixing of surface adatoms into defect sites. In Fig. 1A, the second image shows that the adatom is coalesced into the defect structure. In the following image, it forms a new bond and breaks another bond, resulting in the formation of a new mediator atom. In Fig. 1B, the formation of a mediator atom induces the fast and abrupt structural change, which is explained by our tight-binding molecular dynamics (TBMD) simulation, as shown in fig. S1. When these included adatoms are undercoordinated, it can be considered as mediator atoms and can be seen to catalyze the rapid evolution of defect structure as shown in Fig. 1 (B and C). In Fig. 1C, the existing mediator atom plays a catalytic role of bond formation and breaking. Figure 1C also shows that the undercoordinated mediator atom can be sputtered quickly after its mediating action.

**Fig. 2:**
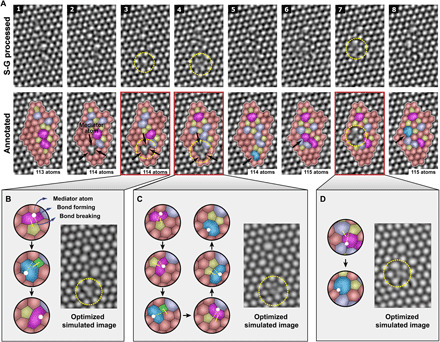
Mediator atom in a complex graphene defect. (**A**) Sequential Savitzky-Golay (S-G) processed AC-TEM images with and without annotations. (**B** to **D**) Analysis of temporally blurred parts in **A-**3, **A-**4, and **A-**7, respectively. The images in (A), (C), and (D) were simulated from DFT-relaxed intermediate stable structures found in mediator atom–induced structural change from TBMD simulations [see movies S1 to S3 for TBMD simulations of (B) to (D), respectively]. The optimized simulated images were obtained by applying convex optimization for the simulated TEM images of the DFT-relaxed structures. The black arrows in (A) show the existence of mediator atoms. The numbers in annotated images are the number of carbon atoms in the area noted by white lines. The optimized simulated TEM images in (B), (C), and (D) are similar to the AC-TEM images in A-3, A-4, and A-7, respectively. The white solid circles in (B) to (D) indicate the positions of mediator atoms. The white dotted lines and solid bars indicate the formation and breakage of bonds to the following structure, respectively. The detailed analysis of other blurry images is shown in fig. S3.

We now study the role of mediator atoms in a more complex graphene defect. Figure 2 shows the generation of mediator atoms and structural change from the combination of a complex defect and an adatom. Because the mediator atom induces large structural change, we analyzed the structural changes by a combination of TBMD, density functional theory (DFT), and TEM image simulations. In the AC-TEM images, the mediator atom can be seen following the inclusion of an additional carbon adatom (Fig. 2A, frames 1 and 2) when we compare the number of carbon atoms between two images. TBMD simulations show that the mediator atoms induce rapid formation and breakage of bonds. We performed TBMD simulations (movies S1 to S3) for the important structural changes and confirmed intermediate stable structures (Fig. 2, B to D) during these structural changes using DFT calculations. The process of sequential structural change in Fig. 2A is also supported by our TBMD simulation (fig. S2). Mediator atom–induced structural changes are found to be responsible for the temporal blurring of the circled part of the AC-TEM images of Fig. 2A (frames 3, 4, and 7), because the TEM image exposure time (3 s) is longer than the transition time. To explain the AC-TEM images, we obtained simulated TEM images for the DFT-relaxed intermediate structures found from the TBMD simulations. Combining simulated images, by taking weights from convex optimization (*19*), results in optimized simulated TEM images that are in excellent agreement with the experimental TEM images as shown in Fig. 2 (B to D). The final structure of the mediator atom–induced structural change in Fig. 2C is the same as the defect structure in Fig. 2A (frame 5). There is also another undercoordinated atom at the bottom of the defect structures in Fig. 2A (frames 6 to 8); however, it is less likely to play a mediating role as the energy barrier (1.7 eV) for it to do so is higher than the energy barrier (0.4 eV) for the proposed mediating role in Fig. 2D (see fig. S3, G to I). In DFT calculations, the mediator atom–induced structural changes in Fig. 2 (B and C) have a series of small energy barriers, the largest of which is 1.2 eV. These are much smaller than the energy barrier for the STW bond rotation in defective graphene (~5 eV).

**Fig. 3:**
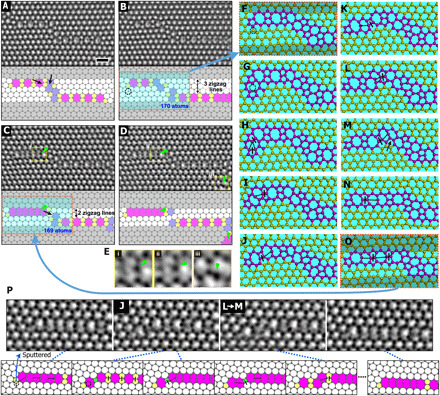
Motion of dislocation kinks induced by mediator atoms in graphene. (**A** to **D**) Sequential Savitzky-Golay processed AC-TEM images and the structural models. (**E**) Enlarged images of yellow dotted boxes in (C) and (D). (**F** to **O**) Snapshots from a TBMD simulation for the structural change from (B) to (C) (see also movie S4). (**P**) Second series of AC-TEM images confirming some intermediate structures (J and L→M) from TBMD simulation and models of the structural change. The solid black arrows indicate the STW-type bond rotations leading to the following image. The green arrowheads in (C) and (D) indicate the existence of mediator atoms in AC-TEM images, with the enlarged images shown in (E). The black dashed circles in (B), (F), (G), and (P) indicate the ejection of an atom. The increase of shaded area from (B) to (C) and from (F) to (O) indicates the jump down of the upper dislocation by one zigzag line. In (H) to (O) and (P), the solid bars and dotted lines indicate the breaking and forming of bonds, respectively, at the following snapshots. In (P), the blue dotted lines indicate the correspondence between the AC-TEM images and the structural models. Scale bar, 0.5 nm.

**Fig. 4:**
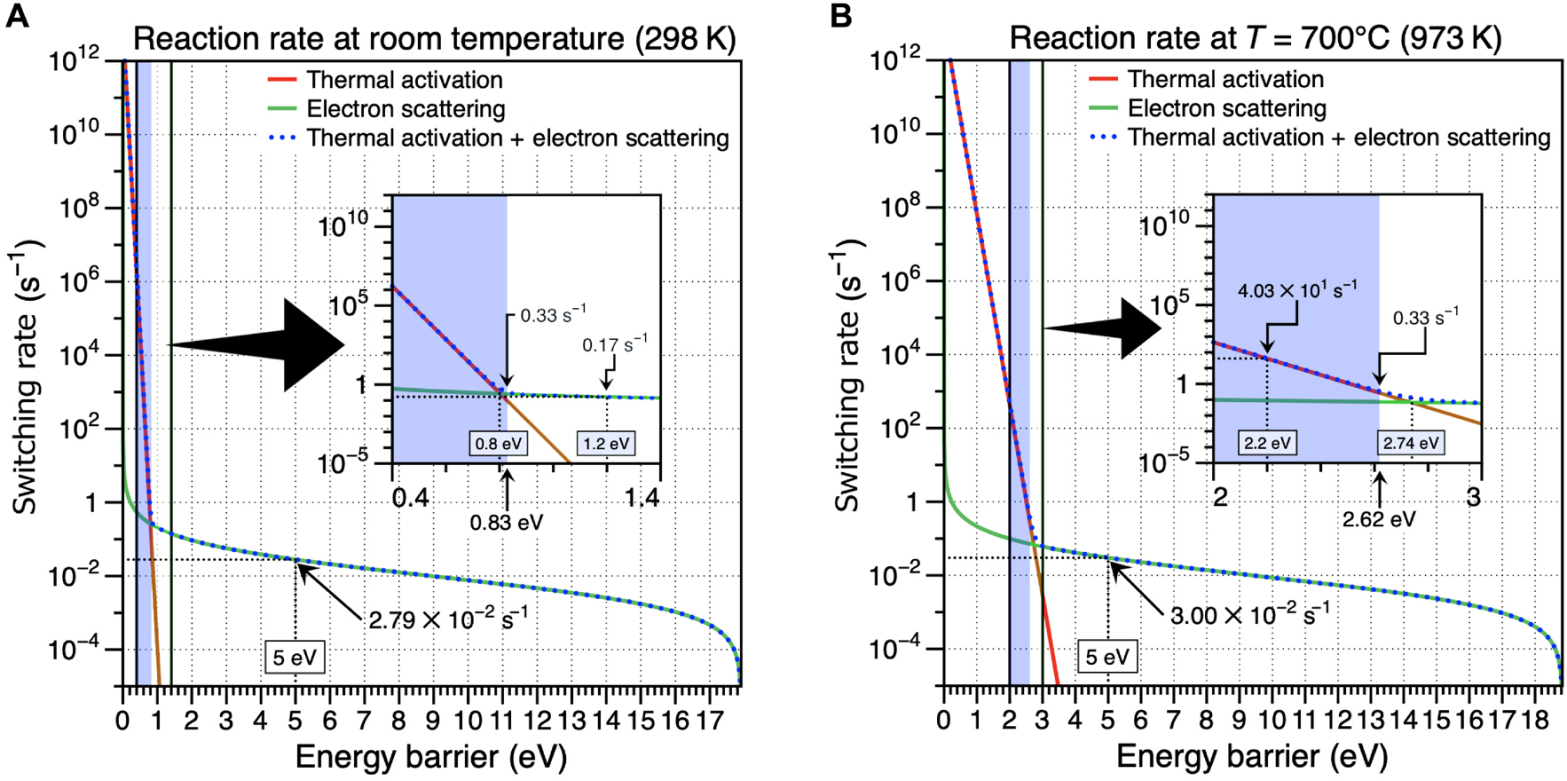
Calculated switching rate curve. (A) at room temperature (298 K) and (B) at 700°C (973 K). The dotted lines show the overall switching rate. The green lines show the contribution from electron scattering (electron dose: 2 × 10^6^ e/s·nm^2^), and the red lines show the contribution from thermal activation. Thermal activation will dominate for processes with energy barrier smaller than 0.8 and 2.74 eV at room temperature and 700°C, respectively. Hence, structural change by STW bond rotation (energy barrier, ~5 eV) is dominated by electron scattering. Blue region (smaller than 0.83 and 2.62 eV at room temperature and 700°C, respectively) indicates that the switching occurs more than once in the exposure time (3 s) and will appear as blurry in TEM images because of the fast structural change. Calculation method and detailed interpretation are shown in discussion S1.

Generation from a vacancy was found to be a second origin for undercoordinated mediator atoms, as can be seen from AC-TEM showing basal dislocation motion in graphene (Fig. 3, A to D), which was performed at high temperature (700°C) using an in situ heating holder. The structural changes from Fig. 3A to 3B and from Fig. 3C to 3D, respectively, can be explained by STW-type bond rotation. However, the structural change from Fig. 3 (B and C) is quite substantial, showing the upper dislocation jumping down by one zigzag line in the time interval (24.0 s) between two images. If we consider only the STW-type bond rotation, the structural change from Fig. 3 (B and C) requires at least five STW-type bond rotations, which we found to be very unlikely to occur in this short time interval from our switching rate calculation (see Fig. 4 and discussion S1). Instead, we consider bond switching induced by mediator atoms. When the structure in Fig. 3C is compared with the structure in Fig. 3B, it can be seen that one carbon atom is missing. The origin of the missing carbon could be due to the diffusion and coalescence of a vacancy or the evaporation of a carbon atom from pentagon and heptagon defects (*20*). We start the TBMD simulation with a vacancy that was allowed to diffuse to the end of the dislocation, which we make by pulling out a carbon atom indicated by a dotted circle in the structural model of Fig. 3B, and then run the TBMD simulation. The generation of this vacancy induces the formation of undercoordinated mediator atoms. After the formation of a mediator atom in the dislocation in Fig. 3G, the breaking and forming of bonds are repeated, as shown in Fig. 3 (G to O), and lastly, the upper dislocation is observed to jump down by one zigzag line in Fig. 3O. In our DFT calculation, the maximum energy barrier for the structural changes from Fig. 3 (G to O) is 2.2 eV, much smaller than the energy barrier (~5 eV) of STW-type bond rotation in graphene nonhexagonal rings. In our TBMD simulation, we find successive and fast bond switching from Fig. 3 (N and O). Such successive and fast bond switching contributes to the average image with many bridging atoms shown in Fig. 3 (C and D) (*21*, *22*). Such an average image and the existence of bridging atoms were reported in our previous study (*2*). In movie S4 from the TBMD simulation results, the role of the mediator atom is clearly shown in the motion of the dislocation. Some of the intermediate structures and translations seen in our TBMD simulations were also observed in other sequential TEM images (Fig. 3P) of graphene partial dislocation glide at room temperature.

**Fig. 5:**
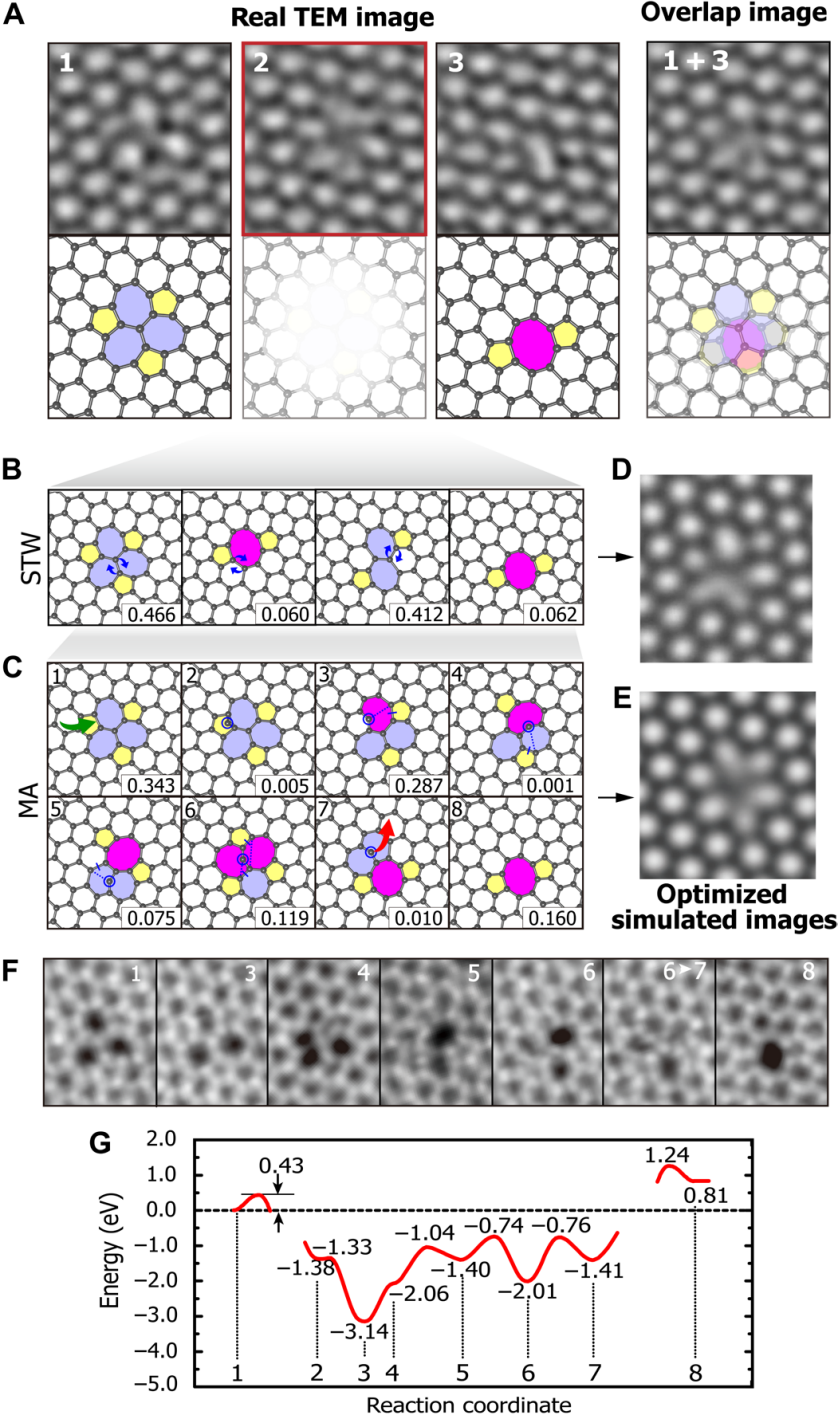
Structural change of divacancy from 555-777 to 5-8-5 in graphene. (**A**) AC-TEM images and the structural models. (1, 555-777; 2, intermediate TEM image; 3, 5-8-5; 1 + 3, overlap of 1 and 3). (**B**) Analysis of the intermediate TEM image by STW-type bond rotation. (**C**) Analysis of the intermediate TEM image by mediator atom (MA) mechanism from TBMD simulation (movie S7). (**D** and **E**) Optimized simulated TEM images from convex optimization for simulated TEM images of structures in (B) and (C). We find best agreement with the real image from the mediator atom simulation (see also fig. S6). (**F**) ADF-STEM images corresponding to the structures in (C). The STEM images are not sequential images but found in various structural changes of divacancy. (**G**) Energy curves for mediator atom mechanism from DFT calculation. The numbers at the bottom of each figure in (B) and (C) are weights of each structure to the optimized simulated image in (D) and (E) obtained from convex optimization. Small circles in (C) indicate the position of mediator atoms. Green and red arrows in (C) indicate the incoming and evaporation of adatom, respectively. The dotted line and solid bar indicate the forming and breaking of bonds, respectively.

Mediator atom–induced structural transformation can be activated either thermally or through electron irradiation during TEM observation. We can compare their relative importance by calculating the switching rate for thermal activation and for electron scattering cross section (*23*–*25*), respectively. Figure 4 (A and B) shows the switching rate curves for those effects at room temperature, where the results of Fig. 2 are obtained, and at 700°C, where the results of Fig. 3 are obtained, respectively (calculation method and detailed interpretation are shown in discussion S1). In Fig. 2, the maximum calculated energy barrier for structural change is 1.2 eV, and most barriers are ~0.8 eV (corresponding switching rate, 0.56 s^−1^ from Fig. 4A) or less, while in Fig. 3, the energy barriers range from 1.4 to 2.2 eV (corresponding switching rate is from 5.6 × 10^5^ to 40 s^−1^ from Fig. 4B). As shown in Fig. 4 (A and B), structural change in these regimes will be dominated by thermal heating rather than electron irradiation. This is why TBMD simulations considering temperature are able to demonstrate the mediator atom mechanism, obtaining intermediate structures during the structural change that match the experiment. In contrast, Fig. 4 (A and B) shows that STW bond rotation activation is hardly affected by thermal heating and instead will be dominated by electron irradiation. However, as the electron irradiation occurs still in our experimental images of mediator atom mechanism under the AC-TEM, it is necessary to consider the effect of such electron irradiation on the mechanism. Therefore, we performed ab initio molecular dynamics (AIMD) simulations considering the role of electron irradiation for the structural changes in Fig. 2A (frames 3 and 7), which is compared with TBMD simulation results (discussion S2). In the simulation, momentum transfer perpendicular to the graphene plane is considered. The results are very similar to high-temperature TBMD results in Fig. 2 (B and D) (see also fig. S4 and movies S5 and S6). In the AIMD simulations, even when the irradiating electron does not directly affect the mediator atom, instead affecting other atoms, the mediator atom mechanism is still observed because of induced lattice vibration and change of strain. This is only possible because the mediator atom–catalyzed energy barrier is substantially lower than for conventional STW bond rotation. Additional DFT calculations of the structural exchange seen in Fig. 2 and the possible electron energy loss spectroscopy (EELS) mapping of scanning TEM (STEM) image series for defect changes related to Fig. 5F exclude the possibility that the mediation involves other elements such as nitrogen or oxygen (discussion S3 and figs. S8 to S10).

The speed at which mediator atom catalysis can occur is also demonstrated with the divacancy, which has been extensively studied as a common defect in graphene (*4*, *6*, *26*). In our AC-TEM data, we occasionally observe intermediate images in the structural changes among divacancy structures, as shown in Fig. 5 and fig. S5. Notably, we observe a structural change of the divacancy in which the 555-777 divacancy, composed of three 5-member rings and three 7-member rings, is changed into the 5-8-5 divacancy, as shown in Fig. 5A. The flower-type image is observed as an intermediate state of the process. The lack of observable atomic structure is because the exposure time is longer than the lifetime of the structural transition states. For the analysis of this intermediate image, we must consider several possibilities. Averaging the before and after images of the sequence in Fig. 5A (“overlap image”) does not agree with the intermediate image, which means that it is not simply an overlay of the initial and final structures but rather contains important information on the structural change. We next produced optimized simulated images using convex optimization analysis to determine whether this change in divacancy was by STW-type bond rotation or by a mediator atom mechanism. Structural transformation via STW-type bond rotation requires four STW-type bond rotations, as shown in Fig. 5B, and the associated optimized simulated TEM image (Fig. 5D) does not exactly coincide with Fig. 5A (frame 2). However, the same procedure using the TBMD simulation intermediates for the mediator atom mechanism (Fig. 5, C and E) is in good agreement. We also compared the similarity among images by using the structural similarity index method (SSIM). The SSIM is a method for measuring the similarity between two images (*27*). The calculated SSIM is 0.91 and 0.73 for the optimized simulated image (Fig. 5E) from the mediator atom mechanism and (Fig. 5D) from the STW mechanism, respectively, compared with the real intermediate TEM image. So, the mediator atom mechanism is closer to the real TEM image compared to the STW mechanism. The trend of weights for the mediator atom–induced intermediate structures (Fig. 5C) obtained from convex optimization is also very similar to that of the DFT-calculated energies (Fig. 5G). A detailed comparison of the four images (Fig. 5A, frame 2; overlap image; simulated STW; simulated mediator atom) is shown in fig. S6. Individual simulated TEM images for the intermediate TBMD structures in the mediator atom mechanism are shown in fig. S7. It is interesting to compare these directly to typical STEM images of a divacancy (Fig. 5F), from various images of a 20-min-long consecutive imaging sequence taken at 500°C. The acquisition time for each scanning image is about 4 s, which is too long to visualize directly all the transformation steps one by one. Nonetheless, the STEM images in Fig. 5F captured in the images at different intervals fit the intermediate transforming status (Fig. 5C) predicted by TBMD. Collectively, the image simulations, DFT calculations, and observations from STEM therefore support the mediator atom mechanism interpretation of our experimental image. The mechanism demonstrated in the TBMD simulations shows that an adatom diffuses and coalesces with the 555-777 divacancy, transforming into a mediator atom and subsequently allowing for fast bond switching (Fig. 5C and movie S7). We also observed other intermediate TEM images in the structural transformation of divacancies (see fig. S5). Repeating the above procedure, when STW and mediator atom mechanisms are considered for the analysis of intermediate images, it is found that the mediator atom mechanism also occurs in a second divacancy transformation (fig. S5A). However, in a third case (fig. S5B), the STW mechanism rather than the mediator atom matches the intermediate TEM image, implying that the STW mechanism can also transform divacancies when mediator atoms are not available. In a fourth example (on-site rotation of 555-777 divacancy in fig. S5C), either mechanism can be interpreted from the image.

## DISCUSSION

We have shown that the undercoordinated atom in graphene defects plays the role of a mediator atom in the breaking and forming of bonds. While it takes a large energy to generate an undercoordinated atom from perfect graphene, it is possible for it to form at defect structures, like grain boundaries or dislocations. While the role of mediator atom mechanism is applied widely in carbon nanomaterials like graphene, because of the stability of sp, sp^2^, and sp^3^ bonds, it can be restricted in other 2D materials. Recently, in the grain boundary of MoS_2_ and WS_2_, it has been reported that the migration of dislocations can be mediated by S vacancies, with the undercoordinated S atom playing the role of a mediator atom breaking and making bonds (*28*, *29*). Even though the mediator atom mechanism is limited in this case, it shows that we can observe the mediator atom mechanism in other 2D materials. A limitation of the mediator atom mechanism is when one mediator atom meets another, which will quench them both. As a simple case, a vacancy with an undercoordinated atom can be filled with diffusing adatoms, saturating the active dangling bonds. However, the mediator atom mechanism can cause notable defect evolution in a very short time due to the low energy barrier. So, the mechanism will be dominant until a quenching event occurs. In this study, we report the catalytic role mediator atoms play in defect evolution by a cooperative study of AC-TEM, annular dark-field (ADF)–STEM, and simulation methods. Mediator atoms induce bond switching with a substantially lower energy barrier compared to the commonly accepted STW-type bond rotation. In graphene defects, we directly confirm the role of the mediator atom and use this to construct a consistent model for the behavior of the mediator atom in other defects. Mediator atoms and their role are also expected to be observed frequently with the development of more advanced microscopy techniques in the future. We contend that the mediator atom will become an important subject of defect study because of its ability to enable alternative low-energy defect evolution pathways.

## MATERIALS AND METHODS

### AC-TEM and ADF-STEM methods

We performed 80-kV AC-TEM on a JEOL 2200MCO with sub-angstrom resolution, achieved by using both aberration correction and double Wien filter monochromation of the electron beam. The AC-TEM for motion of dislocation kinks shown in Fig. 3 (A to D) was performed at high temperature (700°C) using an in situ heating holder. Defects were controllably created by subjecting the graphene to brief doses of high current densities (*30*). Beam current density for imaging is approximately 2 × 10^6^ electrons s^−1^ nm^−2^, and images are taken with exposure time of 3 s at 24-s intervals. Presented TEM images are smoothed with a Savitzky-Golay noise reduction filter, preserving both peak intensity and position.

ADF-STEM images were acquired by using JEOL 2100F–based microscope equipped with dodecaple correctors and a cold field emission gun operating at 60 kV. The probe current was ~30 to 35 pA. The convergence semiangle was 48 mrad, and the inner acquisition semiangle was 53 mrad. STEM images were taken with a size of 256 pixels by 256 pixels and a sample time of 64 μs.

### Simulation methods

#### TBMD simulation

The TBMD simulations were performed using a modified environment-dependent tight-binding (EDTB) carbon potential (*31*), which was modified from the original EDTB carbon potential (*32*) to study carbon sp2 bond networks and has been successfully applied to investigations of various defect structures in graphene (*6*, *33*, *34*). The details of the TBMD simulation methods have been described in previous papers (*31*). The self-consistent calculations are performed by including a Hubbard-*U* term in the tight-binding Hamiltonian to describe correctly charge transfer in carbon atom with dangling bonds and to prevent unrealistic overestimation of charge transfer. The equations of motions are solved by the fifth-order predictor-corrector algorithm with a time step of 1.0 fs. The simulation unit cells are constructed from pristine graphene lattices of 160 carbon atoms for the simulation of the role of mediator atoms in Fig. 2, 480 carbon atoms for the dislocation kink in Fig. 3, and 128 carbon atoms for the structural change of divacancy in Fig. 5. The simulation temperature is kept to 3000 K for the simulation of graphene divacancy and is increased to 3500 K for the defect with mediator atom and to 4000 K for the kink dislocation to accelerate the dynamics, so that structural reconstruction can be observed during the simulation time. Under the electron irradiation in AC-TEM, the creation and transformation of defect structures can originate from energy transfer by electron impact. We check our high-temperature TBMD results by performing AIMD simulation considering the electron impact to the direction perpendicular to graphene plane for the structural changes in Fig. 2 (B and D). It is found that the AIMD simulation results are almost the same as our TBMD simulation results about mediator atom mechanism (fig. S4 and movies S5 and S6). Our TBMD simulations have shown good agreement with many experimental observations under TEM (*2*, *3*, *6*, *35*). Even though our simulation temperature is sometimes above the melting temperature of graphene, the melting is not observed in a short simulation time scale of several tens of picoseconds because the melting is also dependent on the elapsed time. We also used the velocity scaling method to control the temperature.

#### DFT calculation and AIMD simulation

The DFT calculations were performed within the generalized gradient approximation (GGA) of Perdew-Burke-Ernzerhof (PBE) functional (*36*) using Vienna Ab initio simulation package (VASP) code (*37*). In constructing the supercell for simulation, we contain a vacuum region of 30 Å in the *z* direction. One Γ point is sampled as a *k*-point sampling in Brillouin zone. The energy cutoff for plane-wave basis set is 400 eV. When structural relaxations are performed, the structure is fully relaxed until the force on each atom is smaller than 0.02 eV/Å. The AIMD simulations are performed using DFT within GGA, with the PBE functional. After enough steps at room temperature, an energy of 15 eV is transferred to randomly selected atoms in a direction perpendicular to the graphene plane to simulate an electron impact. A time step of 1 fs is found to be sufficient for predicting the dynamics.

#### Image simulation and convex optimization

To obtain the optimized simulated image, we first derive an intermediate structures for the structure transformation obtained by performing the TBMD simulation and perform the DFT calculation on the intermediate structures to obtain the DFT-optimized structures. The image simulations are performed on the DFT-optimized structures to get simulated TEM images. The simulated TEM images are obtained by multislice TEM image simulation with the JEMS software packages (*38*). We combine the simulated images depending on the weights obtained from convex optimization to get an optimized simulated image. The convex optimization method for AC-TEM images is described as follows.

The real AC-TEM image is *A*_0_, and the contributed simulated images are *A*_1_, *A*_2_, …, *A_n_*. They are stored in a form of matrix that contains the pixel values of each location. Each simulated image is weighted by *x*_1_, *x*_2_, …, *x_n_*. Then, our problem is finding the optimized weights *x*_1_, *x*_2_, …, *x_n_* to best fit *A*_0_. To solve the problem, convex optimization is used, which is a mathematical tool that finds the best possible choice of parameter that minimizes the convex objective function (*19*).

To solve our problem, the convex objective function is formulated as ${\left\|A_{0}-\sum_{i=1}^{n}x_{i}A_{i}\right\|}_{F}$, where ‖ ∙ ‖*_F_* is a Frobenius norm of matrix and defined as ${\left\|A\right\|}_{F}=\sqrt{\sum_{i=1}^{m}\sum_{j=1}^{n}{a_{\mathrm{ij}}}^{2}}$. If the pixel values between combined simulated image $\sum_{i=1}^{n}x_{i}A_{i}$ and real AC-TEM image *A*_0_ are similar, then the objective function has a small value. In contrast, if the pixel value difference between the two images is large, the objective function also has a large value. Thus, by minimizing the objective function, we can get the optimal weights *x*_1_, *x*_2_, …, *x_n_* that fits the combined simulated image $\sum_{i=1}^{n}x_{i}A_{i}$ into real AC-TEM image *A*_0_, which is our original goal.

The problem is formulated as follows

$$\text{minimize}\;{\left\|A_{0}-\sum_{i=1}^{n}x_{i}A_{i}\right\|}_{F}$$

$$\text{subject to}\;x_{i}\geq 0,i=1,2,\ldots ,n$$

for a given matrix *A*, the Frobenius norm of matrix *A* is a convex function and defined as

$${\left\|A\right\|}_{F}=\sqrt{\sum_{i=1}^{m}\sum_{j=1}^{n}{a_{\mathrm{ij}}}^{2}}$$

Because the objective function is convex and the feasible set is a convex set, the formulated problem is a convex optimization problem. MATLAB package called CVX (*39*) finds the global minimum solution iteratively, giving the weight values *x*_1_, *x*_2_, …, *x_n_*, the value of objective function ${\left\|A_{0}-\sum_{i=1}^{n}x_{i}A_{i}\right\|}_{F}$, and the optimized simulated image. To measure the quality of obtained weights *x*_1_, *x*_2_, …, *x_n_* and the combined simulated image $\sum_{i=1}^{n}x_{i}A_{i}$, the SSIM (*27*) is used. SSIM measures similarity between two images. SSIM between two image *x* and *y* is defined as

$$\text{SSIM}\;(x,y)=\frac{\left(2\mu_{x}\mu_{y}+c_{1})(2\sigma_{\mathrm{xy}}+c_{2}\right)}{\left(\mu_{x}^{2}+\mu_{y}^{2}+c_{1})(\sigma_{x}^{2}+\sigma_{y}^{2}+c_{2}\right)}$$

where μ means the average of each image, σ means the variance of each image, σ*_xy_* means the correlation coefficient of *x* and *y*, and *c*_1_ and *c*_2_ are constants that stabilizes the division with weak denominator.

## Supplementary Material

aba4942_Movie_S4.mp4

aba4942_Movie_S3.mp4

aba4942_Movie_S6.mp4

aba4942_Movie_S7.mp4

aba4942_Movie_S5.mp4

aba4942_Movie_S2.mp4

aba4942_Movie_S1.mp4

aba4942_SM.pdf

## SUPPLEMENTARY MATERIALS

Supplementary material for this article is available at http://advances.sciencemag.org/cgi/content/full/6/24/eaba4942/DC1

## References

[1] Warner J. H., Margine E. R., Mukai M., Robertson A. W., Giustino F., Kirkland A. I., Dislocation-driven deformations in graphene. Science 337, 209–212 (2012). 22798609 10.1126/science.1217529

[2] Robertson A. W., Lee G.-D., He K., Yoon E., Kirkland A. I., Warner J. H., The role of the bridging atom in stabilizing odd numbered graphene vacancies. Nano Lett. 14, 3972–3980 (2014). 24959991 10.1021/nl501320a

[3] Robertson A. W., Lee G.-D., He K., Yoon E., Kirkland A. I., Warner J. H., Stability and dynamics of the tetravacancy in graphene. Nano Lett. 14, 1634–1642 (2014). 24588782 10.1021/nl500119p

[4] Kotakoski J., Krasheninnikov A. V., Kaiser U., Meyer J. C., From point defects in graphene to two-dimensional amorphous carbon. Phys. Rev. Lett. 106, 105505 (2011). 21469806 10.1103/PhysRevLett.106.105505

[5] Telling R. H., Ewels C. P., El-Barbary A. A., Heggie M. I., Wigner defects bridge the graphite gap. Nat. Mater. 2, 333–337 (2003). 12692535 10.1038/nmat876

[6] Lee G.-D., Wang C. Z., Yoon E., Hwang N.-M., Kim D.-Y., Ho K. M., Diffusion, coalescence, and reconstruction of vacancy defects in graphene layers. Phys. Rev. Lett. 95, 205501 (2005). 16384068 10.1103/PhysRevLett.95.205501

[7] Kim Y., Ihm J., Yoon E., Lee G.-D., Dynamics and stability of divacancy defects in graphene. Phys. Rev. B 84, 075445 (2011).

[8] Kotakoski J., Meyer J. C., Kurasch S., Santos-Cottin D., Kaiser U., Krasheninnikov A. V., Stone-Wales-type transformations in carbon nanostructures driven by electron irradiation. Phys. Rev. B 83, 245420 (2011).

[9] Jeong B. W., Ihm J., Lee G.-D., Stability of dislocation defect with two pentagon-heptagon pairs in graphene. Phys. Rev. B 78, 165403 (2008).

[10] Li L., Reich S., Robertson J., Defect energies of graphite: Density-functional calculations. Phys. Rev. B 72, 184109 (2005).

[11] Eggen B. R., Heggie M. I., Jungnickel G., Latham C. D., Jones R., Briddon P. R., Autocatalysis during fullerene growth. Science 272, 87–90 (1996).

[12] Ewels C. P., Heggie M. I., Briddon P. R., Adatoms and nanoengineering of carbon. Chem. Phys. Lett. 351, 178–182 (2002).

[13] Lee I.-H., Jun S., Kim H., Kim S. Y., Lee Y., Adatom-assisted structural transformations of fullerenes. Appl. Phys. Lett. 88, 011913 (2006).

[14] Jensen P., Gale J., Blase X., Catalysis of nanotube plasticity under tensile strain. Phys. Rev. B 66, 193403 (2002).

[15] Hashimoto A., Suenaga K., Gloter A., Urita K., Iijima S., Direct evidence for atomic defects in graphene layers. Nature 430, 870–873 (2004). 15318216 10.1038/nature02817

[16] Meyer J. C., Girit C. O., Crommie M. F., Zettl A., Imaging and dynamics of light atoms and molecules on graphene. Nature 454, 319–322 (2008). 18633414 10.1038/nature07094

[17] Lehtinen P. O., Foster A. S., Ayuela A., Krasheninnikov A., Nordlund K., Nieminen R. M., Magnetic properties and diffusion of adatoms on a graphene sheet. Phys. Rev. Lett. 91, 017202 (2003). 12906568 10.1103/PhysRevLett.91.017202

[18] Lehtinen P. O., Foster A. S., Ma Y., Krasheninnikov A. V., Nieminen R. M., Irradiation-induced magnetism in graphite: A density functional study. Phys. Rev. Lett. 93, 187202 (2004). 15525202 10.1103/PhysRevLett.93.187202

[19] S. Boyd, L. Vandenberghe, *Convex Optimization* (Cambridge Univ. Press, 2004).

[20] Ding F., Jiao K., Lin Y., Yakobson B. I., How evaporating carbon nanotubes retain their perfection? Nano Lett. 7, 681–684 (2007). 17302460 10.1021/nl0627543

[21] Robertson A. W., Lee G.-D., He K., Fan Y., Allen C. S., Lee S., Kim H., Yoon E., Zheng H., Kirkland A. I., Warner J. H., Partial dislocations in graphene and their atomic level migration dynamics. Nano Lett. 15, 5950–5955 (2015). 26313338 10.1021/acs.nanolett.5b02080

[22] Chen J. H., Autès G., Alem N., Gargiulo F., Gautam A., Linck M., Kisielowski C., Yazyev O. V., Louie S. G., Zettl A., Controlled growth of a line defect in graphene and implications for gate-tunable valley filtering. Phys. Rev. B 89, 121407 (2014).

[23] Zobelli A., Gloter A., Ewels C. P., Seifert G., Colliex C., Electron knock-on cross section of carbon and boron nitride nanotubes. Phys. Rev. B 75, 245402 (2007).

[24] Kim K., Coh S., Kisielowski C., Crommie M. F., Louie S. G., Cohen M. L., Zettl A., Atomically perfect torn graphene edges and their reversible reconstruction. Nat. Commun. 4, 2723 (2013). 24177166 10.1038/ncomms3723

[25] Meyer J. C., Eder F., Kurasch S., Skakalova V., Kotakoski J., Park H. J., Roth S., Chuvilin A., Eyhusen S., Benner G., Krasheninnikov A. V., Kaiser U., Accurate measurement of electron beam induced displacement cross sections for single-layer graphene. Phys. Rev. Lett. 108, 196102 (2012). 23003063 10.1103/PhysRevLett.108.196102

[26] Chen Q., Robertson A. W., He K., Gong C., Yoon E., Lee G.-D., Warner J. H., Atomic level distributed strain within graphene divacancies from bond rotations. ACS Nano 9, 8599–8608 (2015). 26204434 10.1021/acsnano.5b03801

[27] Wang Z., Bovik A. C., Sheikh H. R., Simoncelli E. P., Image quality assessment: From error visibility to structural similarity. IEEE Trans. Image Process. 13, 600–612 (2004). 15376593 10.1109/tip.2003.819861

[28] Azizi A., Zou X., Ercius P., Zhang Z., Elías A. L., Perea-López N., Stone G., Terrones M., Yakobson B. I., Alem N., Dislocation motion and grain boundary migration in two-dimensional tungsten disulphide. Nat. Commun. 5, 4867 (2014). 25202857 10.1038/ncomms5867

[29] Zhou W., Zou X., Najmaei S., Liu Z., Shi Y., Kong J., Lou J., Ajayan P. M., Yakobson B. I., Idrobo J.-C., Intrinsic structural defects in monolayer molybdenum disulfide. Nano Lett. 13, 2615–2622 (2013). 23659662 10.1021/nl4007479

[30] Robertson A. W., Allen C. S., Wu Y. A., He K., Olivier J., Neethling J., Kirkland A. I., Warner J. H., Spatial control of defect creation in graphene at the nanoscale. Nat. Commun. 3, 1144 (2012). 23093181 10.1038/ncomms2141

[31] Lee G.-D., Wang C. Z., Yoon E., Hwang N.-M., Ho K. M., Vacancy defects and the formation of local haeckelite structures in graphene from tight-binding molecular dynamics. Phys. Rev. B 74, 245411 (2006).

[32] Tang M. S., Wang C. Z., Chan C. T., Ho K. M., Environment-dependent tight-binding potential model. Phys. Rev. B 53, 979–982 (1996). 10.1103/physrevb.53.979 9983534

[33] Lee G.-D., Yoon E., Hwang N.-M., Wang C.-Z., Ho K.-M., Formation and development of dislocation in graphene. Appl. Phys. Lett. 102, 021603 (2013).

[34] Lee G.-D., Wang C. Z., Yoon E., Hwang N.-M., Ho K. M., Reconstruction and evaporation at graphene nanoribbon edges. Phys. Rev. B 81, 195419 (2010).

[35] He K., Lee G.-D., Robertson A. W., Yoon E., Warner J. H., Hydrogen-free graphene edges. Nat. Commun. 5, 3040 (2014). 24413607 10.1038/ncomms4040

[36] Perdew J. P., Burke K., Ernzerhof M., Generalized gradient approximation made simple. Phys. Rev. Lett. 77, 3865–3868 (1996). 10062328 10.1103/PhysRevLett.77.3865

[37] Kresse G., Furthmüller J., Efficient iterative schemes for ab initio total-energy calculations using a plane-wave basis set. Phys. Rev. B 54, 11169–11186 (1996). 10.1103/physrevb.54.11169 9984901

[38] Stadelmann P., Image analysis and simulation software in transmission electron microscopy. Microsc. Microanal. 9, 60–61 (2003).

[39] M. Grant, S. Boyd, Y. Ye, *CVX: Matlab Software for Disciplined Convex Programming* (2008).

