# Supplementary material for: Direct observation and catalytic role of mediator atom in 2D materials
Source: Sci Adv. 2020 Jun 10;6(24):eaba4942. doi: 10.1126/sciadv.aba4942 (PMC7286694; doi:10.1126/sciadv.aba4942)
Supplement: aba4942_SM.pdf [file aba4942_SM.pdf]

Science Advances
AAAS

advances.sciencemag.org/cgi/content/full/6/24/eaba4942/DC1

Supplementary Materials for

Direct observation and catalytic role of mediator atom in 2D materials

Gun-Do Lee*, Alex W. Robertson*, Sungwoo Lee, Yung-Chang Lin, Jeong-Wook Oh, Hwanyeol Park, Young-Chang Joo, Euijoon Yoon, Kazu Suenaga, Jamie H. Warner, Christopher P. Ewels*

*Corresponding author. Email: gdlee@snu.ac.kr (G.-D.L.); alex.robertson@materials.ox.ac.uk (A.W.R.), chris.ewels@cnrs-imn.fr (C.P.E.)

Published 10 June 2020, *Sci. Adv.* **6**, eaba4942 (2020)
DOI: 10.1126/sciadv.aba4942

The PDF file includes:

Figs. S1 to S10
Supplementary Discussion 1 to 3

Other Supplementary Material for this manuscript includes the following:

(available at advances.sciencemag.org/cgi/content/full/6/24/eaba4942/DC1)

Movies S1 to S7

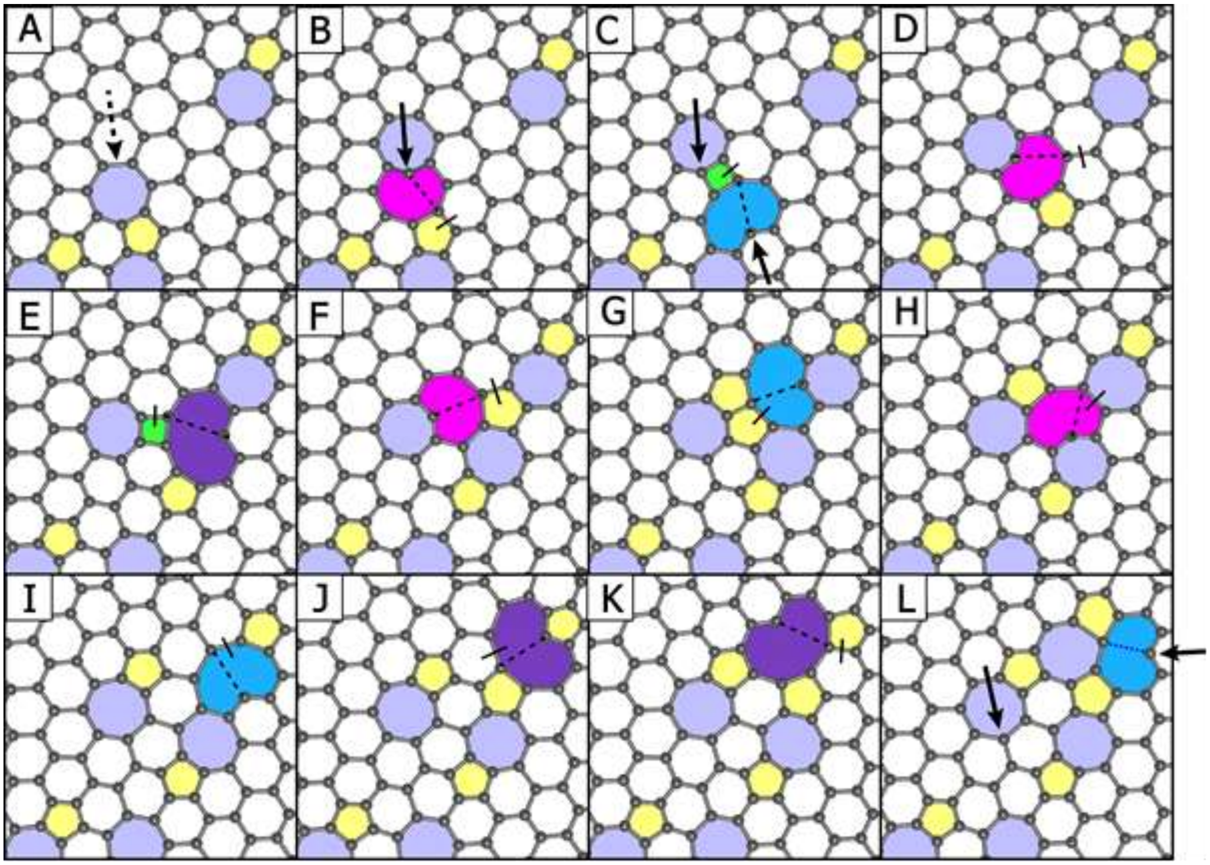

Fig. S1 Atomic model of mediator atom mechanism process suggested by TBMD simulation for the structural change in Fig. 1B. A and **B** corresponding to the first and second TEM images of **Fig. 1B,** respectively. **L** corresponds to the third TEM image of **Fig. 1B.** Red dotted arrows indicate the position at which the adatom will adsorb at the following image. Red arrows indicate the position of adatom. Blue arrows indicate an atom which forms an *sp*^{2} bond and was an adatom before. Green arrows indicate mediator atoms which are newly formed. Dotted lines and solid bars indicate the forming and breaking of bonds, respectively.

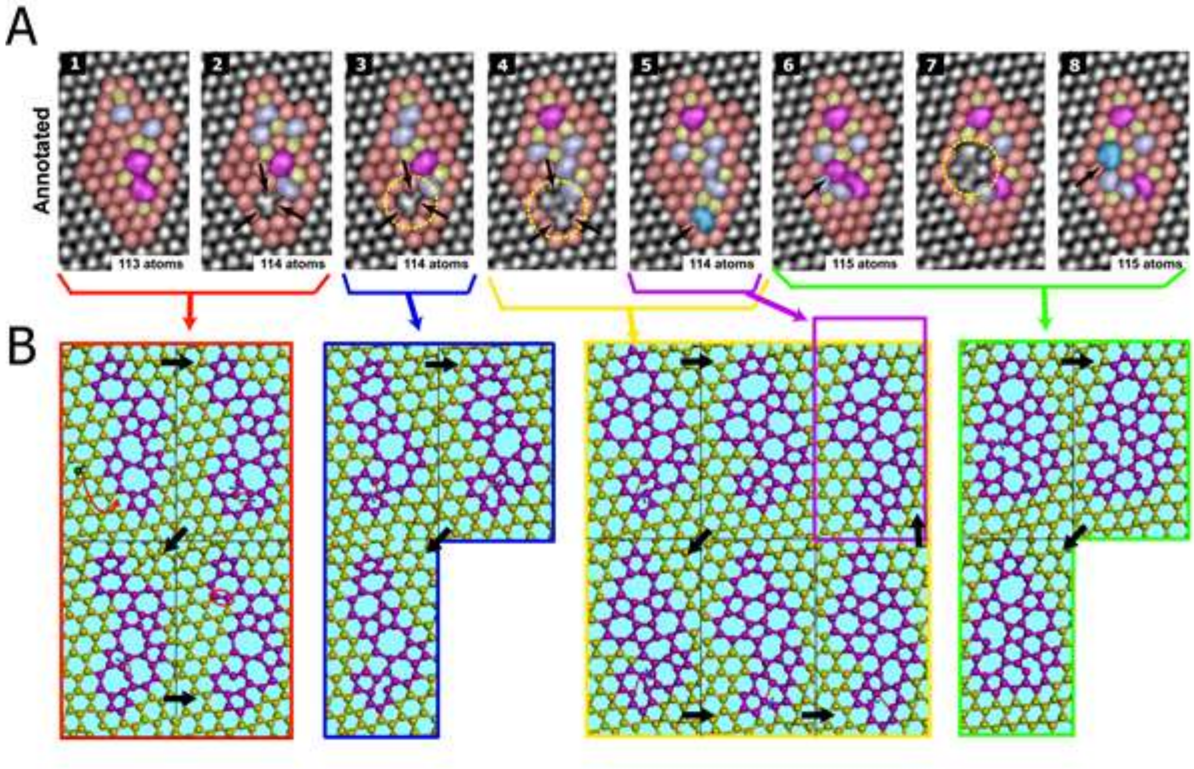

Fig. S2 Snapshots from the TBMD simulation showing the important structural transformation in Fig. 2. A, the annotated image from Fig. 2**A**, **B,** snapshots from the TBMD simulation for the important structural transformation. Thin black arrows in **A** show the existence of mediator atoms. Thick black arrows in **B** indicate the flow of TBMD simulation. Red curved arrow in **B** indicate the inclusion of adatom. Red circles in **B** indicate the STW bond rotation at next step. Blue bars and red dotted lines in **B** indicate the breakage and formation of bonds.

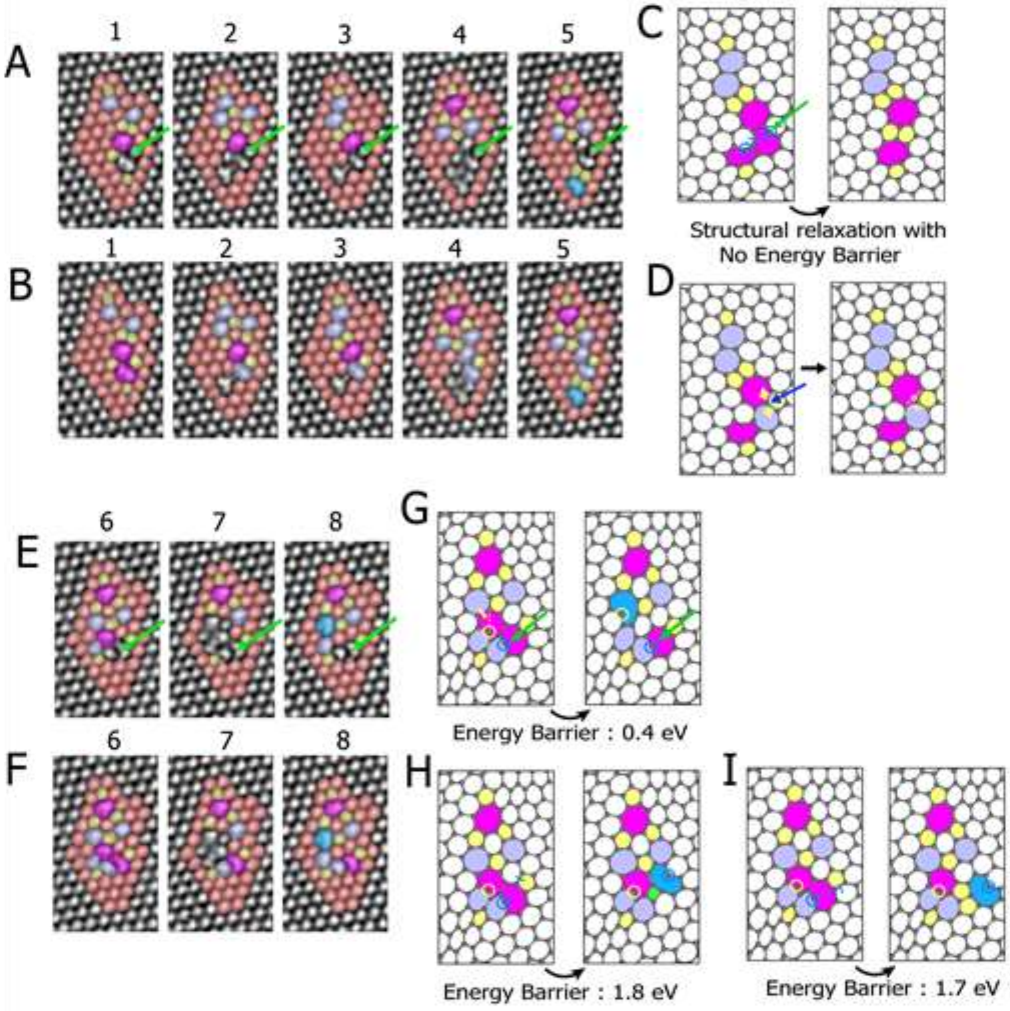

Fig. S3. The analysis of images in initial annotated AC-TEM of Fig. 2. The annotated AC-TEM image in Fig.2 is obtained from the analysis in the present Figure. **A-D**, the analysis of images in fig. S3A, frame1-5, **E-I**, the analysis of images in fig. S3A, frame 6-8, Here, we analyse the blurry images which are not related to mediator atoms mechanism. The analysis of images related to mediator atom mechanism is shown in Fig. 2 of main manuscript. In **A**, we suspect whether there is an under-coordinated atom at same position from 1 to 5 as indicated by green arrows. We put the atom in the structure of 3 as shown in **c** and performed force relaxation by DFT calculation. In this calculation, we found the structure is unstable and changed into other structure without energy barriers as shown in **C**. Therefore, the structure with the under-coordinated atom as shown from 1 to 5 is unstable and could not

be observed in TEM. Therefore, we suggest the structural analysis in **B** for the structures from 1 to 5. We found that the blurry image which can be misunderstood as the existence of under-coordinated atom is due to the softness of bonding with nearby atoms as shown in **D**. When we calculate the energy for the shift from the optimized position of the atom, the energy is lower than the atom in pristine part for the same shift by 1 eV. Therefore, the atom indicated by an blue arrow in **D** is very flexible under electron irradiation and gives blurry image. For the another under-coordinated atom indicated by green arrows in 6, 7, and 8, the energy barriers for its mediating roles is found to be 1.7 eV and 1.8 eV which is much higher (**H** and **I**) than the energy barrier (0.4 eV) of the mediating role (**G**) of mediator atom in yellow circles. So, under the electron irradiation, the atom indicated by yellow circles play a mediating role first as shown in **G** and the atom indicated by an blue circle does not play a mediating role during the observation. Therefore, we determine the structures for 6, 7, and 8 as annotations in **F**. We analyse more the role of mediator atom in Fig. 2 with annotated structures in **B** and **F**.

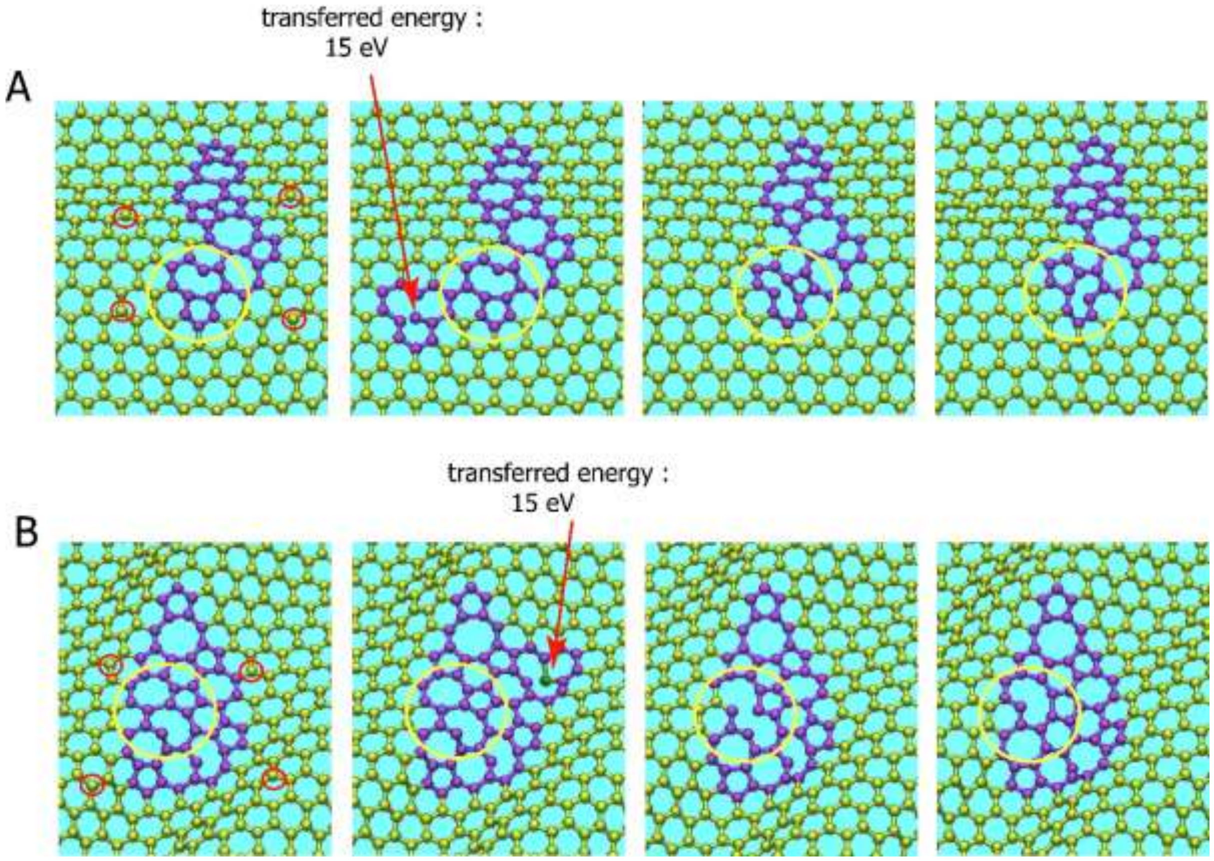

Fig. S4. Snapshots from Ab-initio Molecular Dynamics (AIMD) simulation considering electron irradiation. Energy transfer of 15 eV from electron impact is considered for the perpendicular direction to the graphene plane. **A,** AIMD simulation results for the structural change in Fig. 2A frame 3. It is almost the same as the result from TBMD simulation results in Fig. 2B. **B,** AIMD simulation results for the structural change in Fig. 2A frame 7.It is almost the same as the results from TBMD simulation in Fig. 2D. We considered the electron impact not to mediator atom but to other carbon atoms. We considered the transfer of energy to various atoms as denoted by red small circles in the first figure in **A** and **B** and confirmed the same structural change as shown in the figures. See also Movie S5 for **A** and Movie S6 for **B**. See also Supplementary Discussion 2.

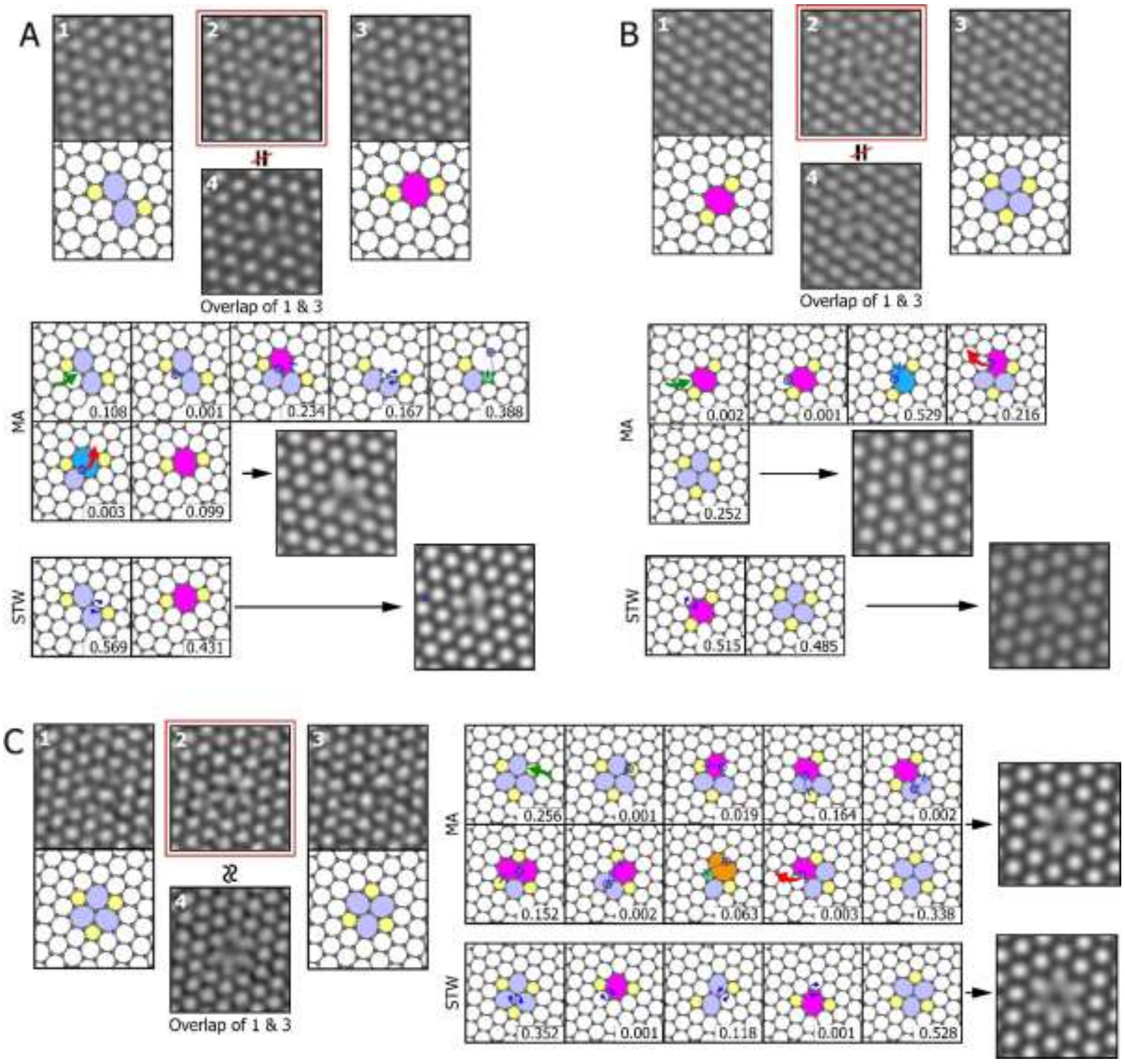

Fig. S5. AC-TEM images during the structural changes of various divacancy structures, the overlap of initial and final AC-TEM images, the analysis of intermediate images and their optimized simulated TEM images from convex optimization for mediator atom (MA) mechanism and Stone-Thrower-Wales (STW) bond rotation. Green and red arrows indicate the inclusion and evaporation of adatoms. The dotted lines and solid bars indicate the formation and breakage of bonds. The small circles indicate the position of mediator atoms. The pair of blue arrows indicated STW bond rotations. **A, Structural change from 5-7-7-5 to 5-8-5,** The overlap image of 1 and 3 is not similar to the intermediate image. In the analysis of intermediate images, the optimized simulated TEM image from MA mechanism is very similar to the intermediate image while the image from STW is different

with the intermediate image. **B, Structural change from 5-8-5 to 555-777,** The overlap image of 1 and 3 is not similar to the intermediate image. However, the optimized simulated TEM image from STW bond rotation is closer to the intermediate image than the image from MA mechanism. **C, Structural change for on-site 60° rotation of 555-777,** The overlap image of 1 and 3 is similar to the intermediate image. The optimized simulated TEM images from both mechanisms are also similar to the intermediate image. Therefore, in this case, it is difficult to distinguish which mechanism is involved.

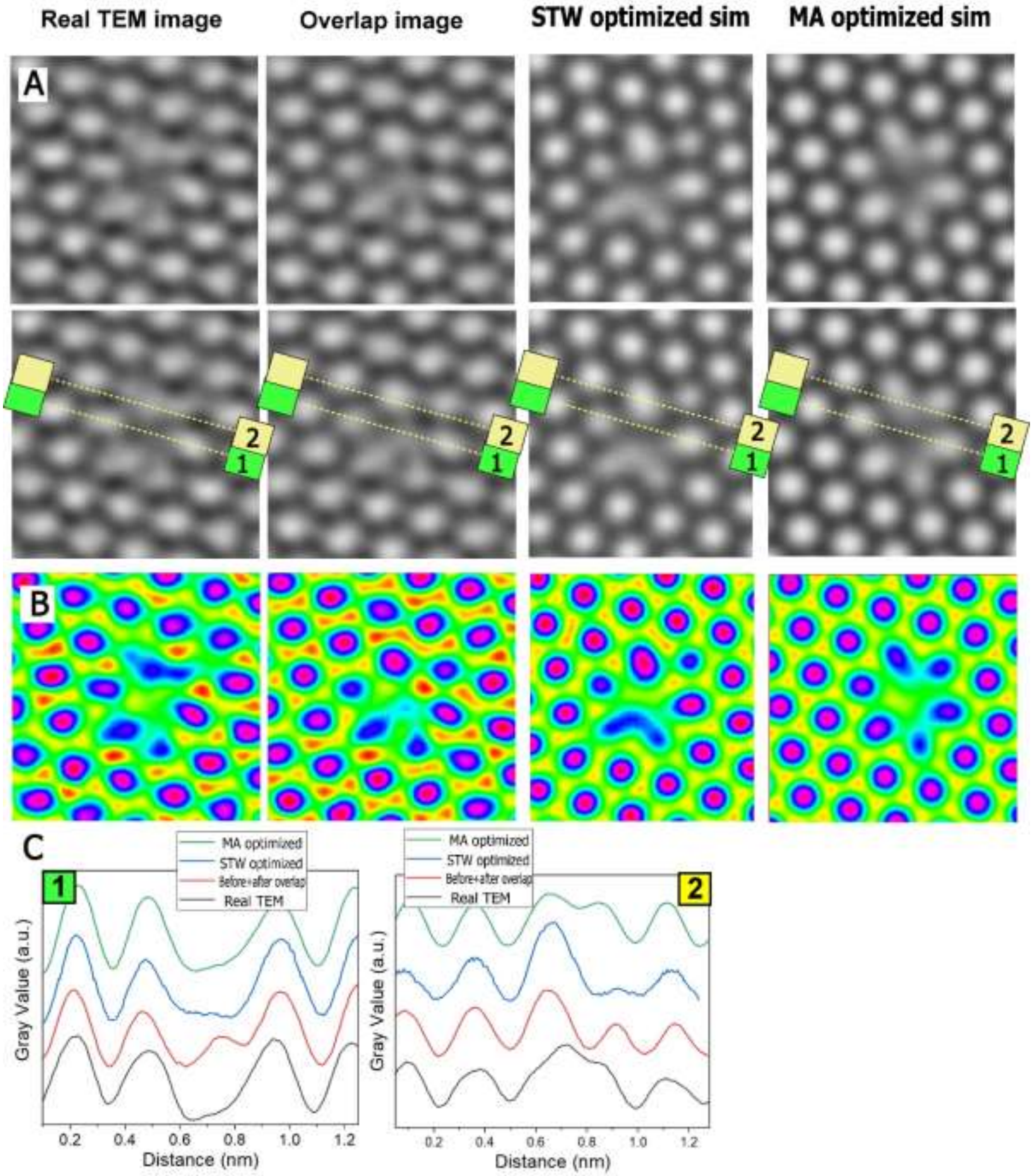

Fig. S6. Comparison of the different possible bond switching mechanisms for the divacancy to the experimentally acquired image. A, The images from Fig. 5 of the main text, showing the intermediate image acquired between a divacancy transition ("Real TEM image"); the output image from simply averaging the before and after image ("Overlap image"); the output from combining the metastable intermediate steps by convex optimization in the STW simulated transition ("STW optimized simulated image"); and from combining the metastable intermediate steps by convex optimization in the

TBMD of the MA simulated transition ("MA optimized simulated image"). **B,** False color versions of the images in **A**. **C,** Intensity profiles acquired across the lines (1) and (2), as indicated by the annotations in **A**. These are acquired along the equivalent axes in all four images, not just the annotated images.

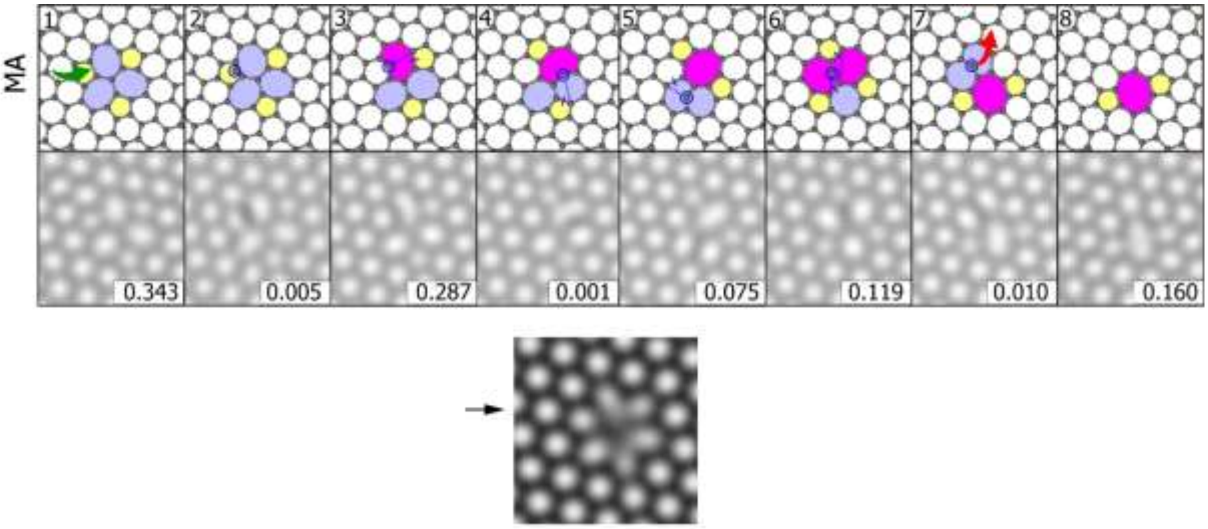

Fig. S7. Simulated images for the structures in the MA mechanism shown in Fig. 5C and the optimized simulated image. The optimized simulated image is obtained by combining simulated images with weights from convex optimization. In the analysis of the simulated images, the structures 3 and 6 as well as the initial and final structures contribute mainly to the optimized simulated TEM image and those structures are also two most stable structures in energy (Fig. 5G) during the structural change.

Supplementary Information Discussion

Supplementary Discussion 1: Calculation of Switching Rate under AC-TEM

1. Switching Rate from Scattering Cross-Section under Electron Irradiation

An analytic approximation of the cross section for Coulomb scattering between an incident electron and a nucleus [24,25] was employed to address the energy transfer rate to carbon atoms during experimental imaging conditions. The scattering cross section for the events when energy E or higher is transferred can be written as

$$\sigma(E) = 4\pi\left(\frac{Ze^2}{4\pi\varepsilon_0 2m_e c^2}\right)^2 \frac{1-\beta^2}{\beta^4}\left\{\frac{E_{\max}}{E} - 1 - \beta^2 \ln\left(\frac{E_{\max}}{E}\right) + \pi\frac{Ze^2}{\hbar c}\beta\left[2\left(\frac{E_{\max}}{E}\right)^{1/2} - \ln\left(\frac{E_{\max}}{E}\right) - 2\right]\right\}$$

where Z is the atomic number of the target atoms, m_e is the electron mass, β = v/c (electron velocity divided by the speed of light c), $E_{\max}$ is the maximum transferred energy in the scattering event. $E_{\max}$ is 15.8 eV for carbon atoms and increases to 17.9 eV [22] and 18.3 eV at room temperature and at 700°C, respectively under the consideration of the effect of thermal lattice vibration. With the experimental imaging condition of electron beam intensity j = 2 × 10^6 e/sec·nm^2, we can calculate the number of scattering events per second as a function of the energy transfer E to single carbon atom.

$$p(E) = -\frac{\mathrm{d}\sigma(E)}{\mathrm{d}E} j$$

Therefore, the switching rate from electron scattering cross-section for the energy barrier (E_b) is calculated as

$$r_{scattering}(E_b) = \int_{E_b}^{E_{max}} p(E)\, dE$$

2. Switching Rate from Thermal Activation

The switching rate from thermal activation is calculated from the first order Arrhenius relation,

$$r_{thermal}(E_b) = \nu e^{-E_b/k_B T}$$

where ν is the attempt frequency, E_b is the energy barrier, k_B is Boltzmann constant, T is temperature. We take here ν to be the Debye frequency, ~10^{13} Hz.

The overall switching rate is determined by the summation of effects ($r_{scattering}(E_b)$ and $r_{thermal}(E_b)$) from electron scattering and thermal activation. When we consider the AC-TEM performed at room temperature (293 K) and 700°C (973 K), the overall switching rate is shown in Fig. 4.

At room temperature, when the barrier height E_b is below 0.8 eV, thermal activation will dominate over electron scattering as shown in Fig. 4a. In Fig. 2 the maximum calculated energy barrier for structural change is 1.2 eV and most barriers are ~ 0.8 eV or less. For individual processes contributing to the overall mechanism to occur within the experimental image exposure time (3 sec), their switching rates must be above 0.33 sec^{-1}. This is marked as a blue region in Fig. 4a, which corresponds to an activation barrier of 0.83 eV or below. Therefore, mediator atom mechanism processes with barriers below 0.83 eV occur faster than the single exposure time and result in the blurry image in our AC-TEM due to the fast structural change. For comparison the energy barrier associated with STW bond rotations (~ 5 eV) will only be e-beam activated and occurs on average 2.79×10^{-2} times per second, *i.e.* the barrier will be overcome once every 36 seconds, much longer than the exposure time.

When we consider the AC-TEM performed at 700 °C (973 K) shown in Fig. 4b, $E_{\max}$ increases to 18.8

eV. At this temperature, for energy barrier less than 2.74 eV, thermal activation will dominate over electron scattering. The energy barriers for kink motion of dislocations in graphene are below 2.20 eV from our DFT calculations, and therefore at this temperature thermal activation will dominate the kink motion. From the calculation of switching rate, hopping events will occur faster than 40 times per second. This means that over a single image experimental exposure time (3 sec), there is sufficient time for all the processes from Fig. 3b to Fig. 3c to occur. In this way we can explain the abrupt change of dislocation kink in AC-TEM as shown in Fig. 3. For the comparison, the STW bond rotation (energy barrier: ~ 5eV) occurs on average 3.00×10^{-2} times per second at 700 °C, *i.e.* the barrier will be overcome once every 33 seconds. However, as we mentioned in the main text, the structural change from Fig. 3b to 3c requires at least five STW type bond rotations, which will take over 150 seconds. It is much longer than the time interval (24.0 sec) between two images. That's the reason why the abrupt change of dislocation kinks in such a time interval cannot be explained by the STW bond rotation.

24 Kim, K. et al. Atomically perfect torn graphene edges and their reversible reconstruction. Nat Commun 4, doi:10.1038/ncomms3723 (2013).
25 Meyer, J. C. et al. Accurate Measurement of Electron Beam Induced Displacement Cross Sections for Single-Layer Graphene. Phys Rev Lett 108, 196102, doi:10.1103/PhysRevLett.108.196102 (2012).

Supplementary Discussion 2 : Consideration of Electron Impact under AC-TEM

In this discussion, we consider the electron impact under AC-TEM. As we mention in Supplementary Discussion 1 about the switching rate theory from electron scattering cross section and thermal activation, much of mediator atom mechanism belongs to the region governed by thermal activation rather than the effect of electron irradiation. However, since our TEM images are acquired from AC-TEM, we need to consider the electron impact. In order to study the electron impact, we have performed ab initio molecular dynamics simulation (AIMD) simulation considering momentum transfer corresponding to energy 15 keV to perpendicular direction to graphene plane. We choose various atoms as the objective atom of momentum transfer as denoted by red circles in fig. S4 and it is found that the AIMD results are almost the same as our TBMD results obtained by thermal activation. Especially, we performed it for the structural changes shown in Fig. 2B and 2D (See fig. S4, Movie S5 and Movie S6)

and it shows almost same results as shown in Fig. 2B and 2D (See also Movie S1 and S3 in our manuscript). From the Movie S5 and S6, we can find that the mediator atom mechanism can be observed by the fluctuation of the graphene plane or by the change of strain even in the case of electron impact to carbon atoms which are not mediator atoms. Therefore, in case of a high energy barrier of 5 eV, which corresponds to the STW bond rotation, the electron irradiation situation should be considered. However, the mediator atom mechanism, which corresponds to an energy barrier below 0.8 eV at room temperature (energy barrier below 2.2 eV at 700 °C), can be sufficient for thermal activation regardless of electron irradiation conditions. Therefore, in the analysis of mediator atom mechanism, the result considering electron irradiation is not significantly different from the result of thermal activation.

Supplementary Discussion 3: Discussion about the Possibility of Other Atoms (N or O) for Mediator Atom

As an under-coordinated atom, the mediator atom usually bonds with two carbon atoms. We may suspect the mediator atoms as N or O atoms because those atoms can form bonds with two carbon atoms. Here, we would like to discuss the possibility of N or O atoms for mediator atoms in Fig. 2A frame 2, frame 6 and frame 8. There is no mediator atom in Fig. 2A frame 1 and the number of carbon atoms in the white line is 113 as shown in Fig. 2. We consider all atoms in Fig. 2A frame 1 as carbon atoms because there is no under-coordinated atom. As shown in Fig. 2A frame 2, one atom is added so that the number of atoms in the white line increases to 114. One added atom in Fig. 2A frame 2 can be assumed as N (or O) atom. In this case, the other two under-coordinated atoms will be carbon atoms because three under-coordinated atoms are found in Fig. 2A frame 2. When we perform the DFT calculation for the structure, two carbon atoms always make a bond to each other as shown in fig. S8 (Although fig. S8 is studied for the analysis of Fig. 2A frame 3, the important part noted by dotted yellow circles is same as that in Fig. 2A frame 2) and the structural change does not occur likely to that of Fig. 2B. Therefore, the simulated TEM image is not same as Fig. 2A frame 2 as shown in fig. S8. We can also consider the case that three under-coordinated atoms are all N (or O) atoms. In this case, the simulated TEM images can be same as Fig. 2A frame 2 but two carbon atoms should be replaced by two incoming N atoms

because only one N atom can be added from Fig. 2A frame 1 to Fig. 2A frame 2. However, the replacement is difficult because two carbon atoms should be evaporated first and two N atoms should diffuse in. Such a complex mechanism is almost impossible in one exposure time of 80 kV TEM if N or O rich condition is not intentionally made. Even if the replacement happens and three under-coordinated atoms become all N atoms, the structure of three N atoms are quite stable and the structure cannot be changed into the structure in Fig. 2A frame 4 because at least two N atoms should be replaced by two carbon atoms again on the way to the structure of Fig. 2A frame 4.

We also consider the possibility of N (or O) atoms as the under-coordinated atoms in Fig. 2A frame 6 and frame 8. For the structural change from frame 6 to frame 8, we can consider the most favorable pathway (fig. S9) from the DFT search of various pathways. Because the energy barriers (2.5eV ~ 3.5 eV) for the process are very high compared to mediator atom mechanism by carbon atoms, it is very difficult to achieve the complex process in a short exposure time. When we consider the switching rate in Fig. 4, the process takes over 10 sec which was longer than one exposure time (3 sec). Furthermore, the process of the structural change cannot explain the TEM image as shown in fig. S9. Therefore, the possibility that the under-coordinated atoms can be N or O atoms in Fig. 2 is excluded. Overall, when nitrogen or oxygen atoms are present, it is fairly stable and the structural change does not occur in a short time, resulting in no blurry image from the rapid structural change. The structural change involved by N or O atom cannot explain the intermediate images in our TEM.

We also performed electron energy loss spectroscopy (EELS) for some images in STEM image series of defect change containing STEM images in Fig. 5F. The EELS mapping for those STEM images is shown in fig. S10. In this EELS mapping, we did not find any signal from other element such as N or O.

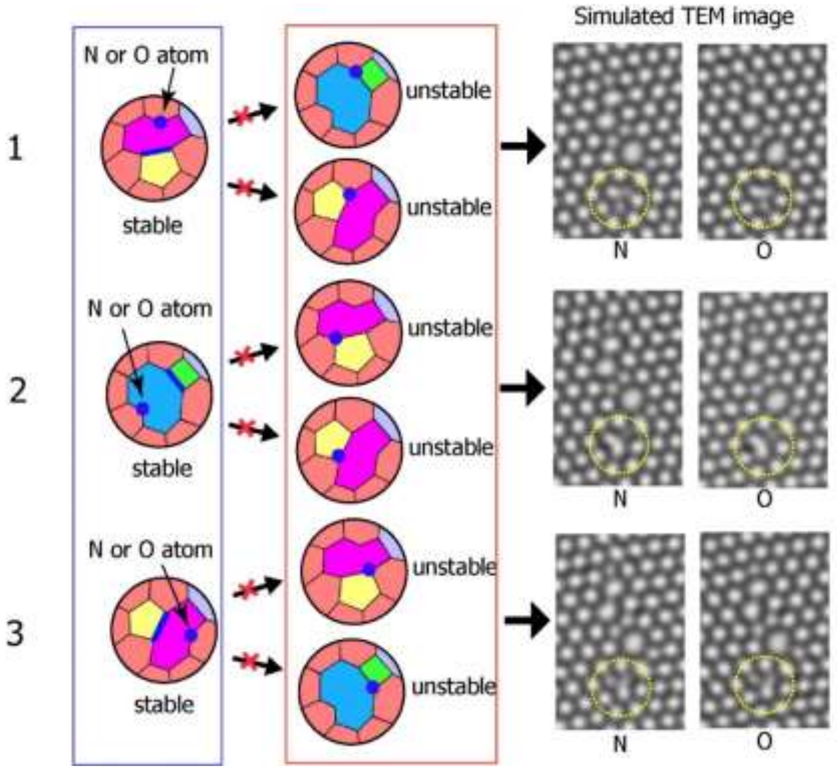

Fig. S8, DFT calculated structures and corresponding simulated TEM images in the case that one of under-coordinated atoms in Fig. 2A frame 3 is N or O atom.

In Fig. 2A frame 3, if one of three under-coordinated atoms is assumed to be a N or O atom, other two carbon atoms always make a bond to each other and the structural change does not occur likely to that of Fig. 2B because the structures in the red square box are unstable and cannot form in our DFT calculation. The simulated TEM images are not same as Fig. 2A frame 2 and 2A frame 3.

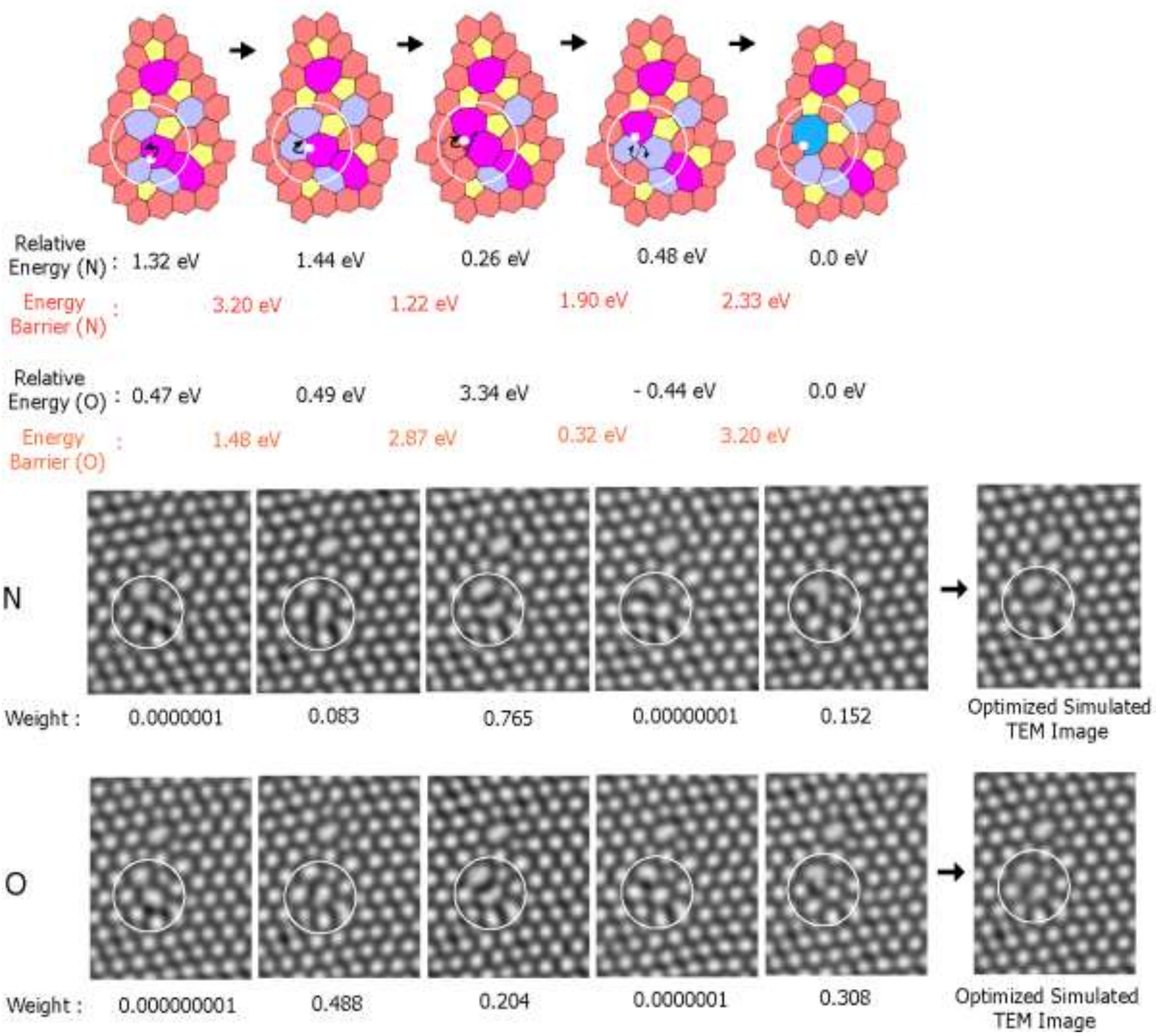

Fig. S9, DFT and convex optimization results showing the exclusion of possibility that the mediation involves O or N. DFT calculated structural change, corresponding energy (energy barrier), and simulated TEM images for the structural changes and optimized simulated TEM images by convex optimization in the case that one of under-coordinated atoms in Fig. 2A frame 6 and frame 8 is N or O atom. The optimized simulated TEM images are not in agreement with the intermediate TEM image in Fig. 2A frame 7.

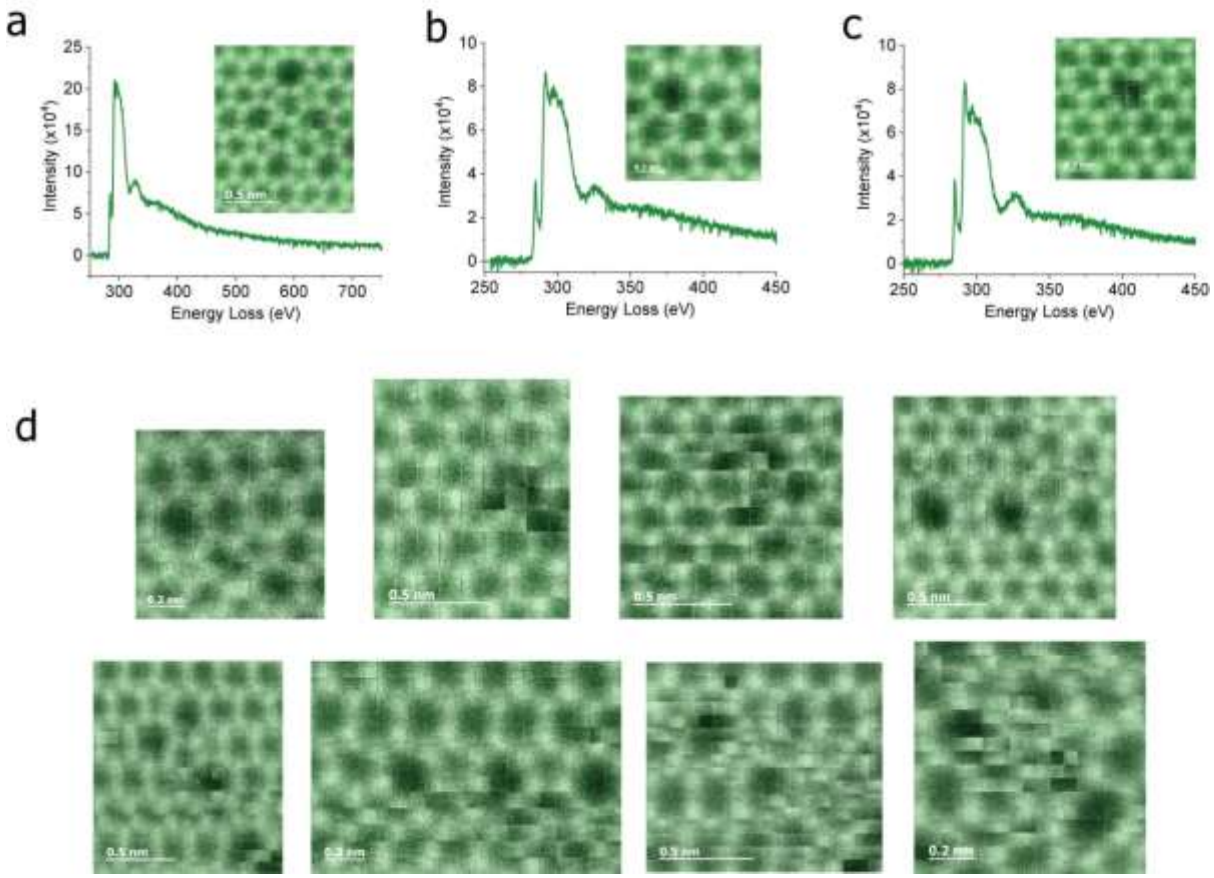

Fig. S10, A-C, EELS color mapping and spectra on graphene defects, D, EELS color mapping of various graphene defects. Green color represent the EELS signal of carbon (selected energy range: 280~350 eV). No Nitrogen (~400 eV) and Oxygen (~540 eV) signal was found in the EELS mapping area. The STEM images in Fig. 5F are parts of the STEM image series containing STEM images of **A-D**.
